# A robust and dynamic malware detection and classification model using behavioral-based analysis and BERT technique

**DOI:** 10.1371/journal.pone.0327604

**Published:** 2025-09-04

**Authors:** Abdulrahman Hassan Alhazmi

**Affiliations:** Department of Computer Science, College of Engineering and Computer Science, Jazan University, Jazan, Kingdom of Saudi Arabia; University of the West of Scotland, UNITED KINGDOM OF GREAT BRITAIN AND NORTHERN IRELAND

## Abstract

Malware classification is a challenging task due to the constantly evolving nature of malicious software. Traditional signature-based methods and static analysis often fail to detect sophisticated threats, making behavior-based analysis crucial. This study proposes a malware detection model that analyzes the behavior of executable files (.exe) to classify them as malware. The model submits the file to VirusTotal, where it runs in a secure environment to monitor actions such as file modifications, registry changes, or network connections. To enhance detection accuracy, the BERT model is applied to extract key features from these behavior logs. After 100 training epochs, the model achieved 92.25% accuracy and an F1-score of 91.22%, demonstrating strong overall performance. Class-wise evaluation was also conducted, treating each malware family as a distinct class to assess specific detection accuracy. Furthermore, a correlation matrix was analyzed to explore inter-class relationships and identify overlapping behaviors. Experimental results show that SVM achieved the highest F1-Scores for Adware (0.98) and BackDoor (0.91), while Random Forest showed comparable performance. Naïve Bayes, however, performed poorly for FakeAlert (F1-Score: 0.64). These findings confirm the effectiveness of the proposed behavior-based approach using BERT features, with SVM and Random Forest proving to be the most reliable classifiers.

## 1. Introduction

**Fig 1:**
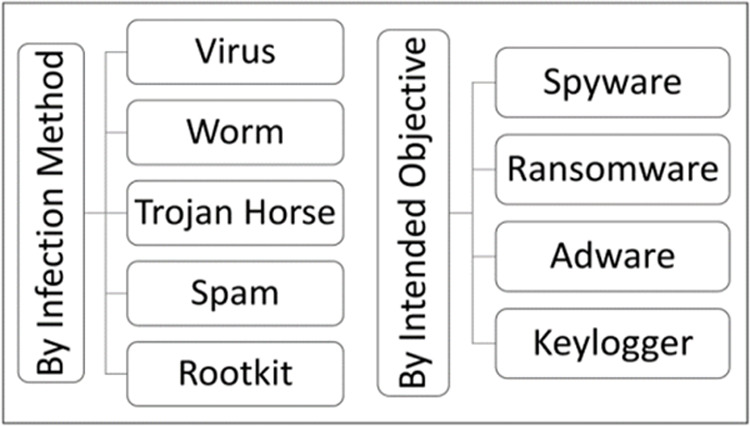
Classification of Malware [1].

The world has taken a new direction due to extensive use of computing technology especially Internet of Things devices. These technologies create a multitude of security risks because of inadequate management as well as user’s ignorance of security [1]. Proliferation of digital services has increased the threat of malicious software. It has also made it difficult to identify different kinds of malware such as Adware, viruses, Spyware, Botnets, Worms, and Ransomware [2]. Different types of malwares are shown in Fig 1.

Malware refers to any piece of software that is designed to deliberately carry out harmful payloads on target equipment. These victim devices can include computing devices, computer networks, smart phones and other similar smart devices [3]. Malware poses several detrimental risks to computers, such as system damage, remote execution of malicious code, and theft, corruption, altering, or deletion of important data. The stolen data can be utilized for many harmful purposes, resulting in significant harm. The weak security measures of sensors connected to wireless networks make them susceptible to malware assaults [4,5].

According to projections, the yearly worldwide expenses of cybercrime are expected to increase significantly from $3 trillion in 2015 to $10.5 trillion by 2025 [6,7]. Among different cyber-attacks, more than 50% are based on malware attacks. Malware analysis is based on static, dynamic and hybrid approaches. In static approach, analysis includes analyzing the structure without running the executable file. Structuring and analyzing the binary code allow getting more specific information on how the virus works. Static analysis requires no execution and uses less processing time and becomes resource efficient [8,9]. In contrast to static analysis, dynamic approach executes the malware file to determine its behavior. It helps in detection of malicious activities on the runtime, where malicious files are executed on virtual machine under controlled conditions. Dynamic analysis offers significant benefits due to its automated nature, allowing for its use on a broad scale as long as there are the resources available [7]. But some malwares have capability to change their way of working or start working like normal programs after identifying virtual machines. It becomes one of the serious concerns while detecting malicious software using this approach and required to make sure that emulator remains identical. Sometimes, virus remains dormant due to which their behavior cannot be identified. There is a potential danger of malware infection and its subsequent dissemination if the virtual environment is not adequately regulated [10,11].

Dynamic malware analysis facilitates to visualize and predict the analysis of function parameters, enables the monitoring of function calls and tracking of the flow of instructions. It allows memory analysis as well in which it mostly relies on the quantity and features of characteristics of Windows API calls, as well as registry alterations, modifications of file system, and data flow in network. Furthermore, a comparison of system before and after malware execution might provide valuable insights on the impact produced by the malware [12].

## 2. Contribution

The contribution of proposed technique is as under:

- Malware behavior analysis data creation is important step for classification malware in different classes, for this purposed malware behavior data is generated from virus total online library.
- Traditional NLP based machine learning algorithms used natural text data; this article introduces a novel method by applying the BERT model for the classification of malware based on behavior logs. BERT’s bidirectional context makes it easy to understand complex malware actions, such as network activity, API calls, and system processes, for more accurate classification.
- By extending BERT’s powerful contextual learning capabilities, the approach enhances the accuracy of malware detection and classification, outperforming traditional methods.

## 3. Literature review

Malware refers to harmful code designed to cause damage. Its negative effects commonly include, but are not limited to, information confidentiality breaches, impairing data integrity, stealing information, and causing denial of service. Malware can perform various operations on a target system or network, often without the owner’s knowledge. These operations include file manipulation (reading, writing, deleting, modifying, and moving files), starting and stopping services, creating, modifying, or deleting registry values, creating or deleting mutexes, managing runtime dynamic link libraries (DLLs), and executing network functions such as communication ports and transport protocols [13]. The study [14] presents a new hybrid method to improve IP reputation systems by combining Cyber Threat Intelligence, Dynamic Malware Analysis, Data Forensics, and Machine Learning. The goal of this approach is to detect malign IP addresses and zero-day attacks before any communication takes place. It addresses the drawbacks of current systems, such as high code for management resources and false positives. The method utilizes big data forensics to forecast IP reputation, enhance detection precision, and evaluate risk. Evaluations demonstrate that the proposed system surpasses the traditional reputation systems in terms of accuracy, recall, and overall security efficacy [15].

The research is centered around the detection of dynamic malware, with a particular emphasis on the requirement for effective methods to deal with the fast-changing nature of malicious code that takes advantage of internet vulnerabilities. The ineffectiveness of conventional and heuristic malware analysis has resulted in the adoption of automated, behavior-based detection techniques that employ machine learning (ML). The study [16] obtained good accuracy in the detection of malware by utilizing a variety of classifiers, such as extra trees, RF, and Gaussian NB SGD. The study emphasis the significance of reducing the number of features used for detection and improving machine learning models to manage the complexity of malware. The models were trained and tested using a dataset from Kaggle that included 531 features and 373 samples. According to the research, ensemble deep learning models have drastically boost detection accuracy. Therefore, it is recommended that future works concentrate on the optimization of feature selection and model performance in order to increase malware detection rates, decrease false positives, and expedite the detection process.

The another study [17] focuses on improving ransom ware detection using ML and dynamic analysis. It develops a novel dataset of dynamic features obtained from both ransom ware (locker variants and encryptor) and good ware, and then uses ML techniques to identify ransom ware with high accuracy. Experiments were performed on five platforms using 20 ransom ware and 20 good ware samples, yielding a dataset of 2000 records containing 50 chosen attributes. The models, which included gradient boosted regression trees, random forest, and neural networks, produced virtually flawless detection rates while dramatically increasing processing speeds and accuracy over previous techniques. The completed dataset is publicly available to enable further study and progress in ransom ware detection. This work addresses the problem of extracting features from such evasive malware which can mask its true behavior at analysis time and makes the traditional feature extraction useless. They introduce a new methodology of Dynamic Initial Evasion Behaviors Determination (DIEBD) [18]; using this approach entropy analysis is performed on enhanced features with API-grams, in order to monitor and identify typical evasion behaviors. It outperformed previous automatic methods resulting in accuracy of 96.7%, and F1-score = 0.975 using the Xgboost classifier. However, this method has limitations that it only detects partial evasion behaviors and does not identify some later-evasion techniques in malware lifecycle. Future research will investigate evasion tendencies without API calls and how to distinguish between benign and malicious evasions.

The study presents in [19] an approach that uses multi-edge directed heterogeneous graphs developed from API calls to depict executable behavior in order to identify zero-day malware in Windows PE files. In comparison to current approaches, a graph attention network is applied to these networks in order to evaluate the significance specific behavior, improving malware detection accuracy and false positive rates. Despite promising results, it has drawbacks like dependence on sandbox conditions and difficulty in maintaining model efficacy because of the continuous evolution of malware. On the basis of Explainable AI along with dynamic analysis, XRan is an advanced ransom malware detection system. It validates and trains with the integrated sequences of API calls, DLLs using CNN, and Mutual Exclusions. Merging these features allows XRan to use SHAP and LIME in providing improved accuracy of detection and transparency in conclusions. It surpasses current techniques with a True Positive Rate (TPR) of up to 99.4% and provides comprehensive insights into the detecting process [20]. The research introduces a hybrid model that merges hard voting as a meta-learner with Support Vector Machine (SVM), Logistic Regression (LR) and KNN algorithms for handling the growing complexity of malware. The goal of this methodology is to identify emerging malware effectively and correctly. With Avgsig and Windows PE files as source datasets, the model obtained 99.7% accuracy and 99% F-score, with KNN exhibiting the greatest performance alone. The low error rate of 0.30% and fast running time of the findings outperform existing methods. Future research will focus on unsupervised and deep learning techniques to further improve malware detection and efficiency [21]. Table 1 shows overall comparison of the different malware classification stat-or-art methods.

**Table 1:** Performance comparison of state-of-art malware classification existing techniques.

Ref.	Year	Features	Dataset	Models Used	RESULTS/ Accuracy (%)	Limitations
[15]	2021	i. Flow-level features (like packet counts, durations) ii. Packet-level features (header information)	i NSL-KDD ii UNSW-NB15 iii Custom network captures	i Convolutional Neural Networks (CNN) ii Long Short-Term Memory networks (LSTM)	–	i Dependency on labelled data ii High computational resource requirements
[16]	2023	531 features extracted from Windows programs	Dataset from Kaggle included 373 samples, with 301 being malicious and 72 safe	i RF ii SG iii Extra trees iv Gaussian NB	100%	Requirement for i Optimization of feature selection ii Improvement of model generalization for real-world applicability.
[17]	2023	50 dynamic features related to ransom ware behavior	2000 records from 20 ransom ware and 20 good ware samples, generated across five platforms	i Gradient boosted regression trees ii Random Forest iii Neural networks	i Neural Networks: 98.06% ii Random Forest: 92.88% iii Gradient Boosted Trees: 99.68%	i High processing times in initial stages ii Further testing with broader datasets
[18]	2023	i Representative evasion behaviors ii Dynamic Initial Evasion Behaviours Determination (DIEBD) technique iii Entropy variations in API-gram features	Dataset consists of benign and evasive malware samples	Xgboost classifier	96.7%	Model cannot detect behaviours occurring later in the malware’s lifecycle or without API calls.
[19]	2024	Multi-edge directional heterogeneous graphs constructed from API calls	Data generated from Cuckoo sandbox	Neural network-based DL model using graph attention network	The model’s accuracy was not the highest but showed competitive results against leading detection engines.	i Dependency on a sandbox environment ii Issues with anti-virtualization techniques
[20]	2024	XRan integrates sequences of i API calls ii DLLs iii Mutexes to enhance ransom ware detection	5 diverse datasets, including 6,263 crypto-ransom ware samples	CNN with Explainable AI models (LIME and SHAP)	99.4%	Generalization to other ransom ware types or newer threats may need further validation
[21]	2024	Numerical data and malware characteristics	i Avgsig with 20,000 entries ii Windows PE files with 19,611 samples	Combines i KNN ii SVM iii LR with hard voting	i Ensemble Voting classifier: 99.7% ii SVM: 98.7% iii KNN: 99.8% iv LR: 49.66%	Logistic Regression performed poorly as compared to other models
[22]	2024	Numerical Data (Permissions count, Smali size, Permission rate)	10,000 Android Apps (Self-collected)	SVM, RF, Rotation Forest, NB	Optimized up to 89.96% of features	Focus on permission-based features only, No comparison with BERT-based methods
[23]	2024	Numerical Data (Dynamic and Hybrid Features)	Hybrid analysis-based dataset (Real device execution, Apps from 2008–2020)	RF, LR, DT, SVM	97.86%	No use of deep learning or BERT-based models, limited to classical ML

## 4. Proposed model

**Fig 2:**
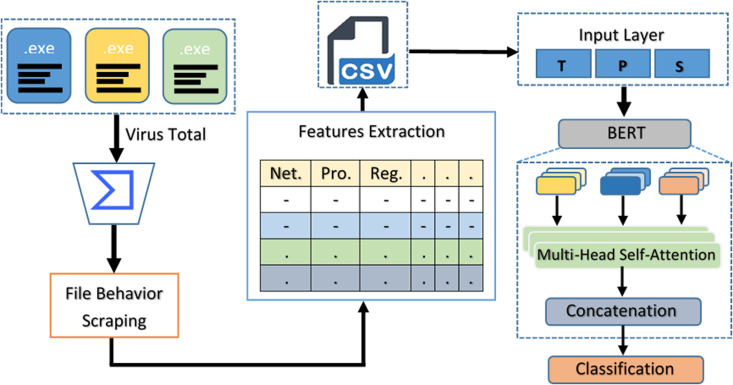
Flow Diagram of Proposed model.

The proposed model works by analyzing the behavior of executable files (.exe) to detect malware. First, the file is submitted to VirusTotal, which runs it in a safe environment and tracks its actions, like modifying files, changing registry settings, or making network connections. This behavioral data gives a detailed view of how the file behaves. Then the BERT model is used, which is typically meant for understanding text, to pick out important features from the behavior logs. These features help us identify patterns that might indicate whether the file is harmful or not. Finally, using this information, the file is either classified as malware or safe software, helping to improve malware detection accuracy. Proposed model diagram in shown in Fig 2.

### 4.1. Data preparation

**Fig 3:**
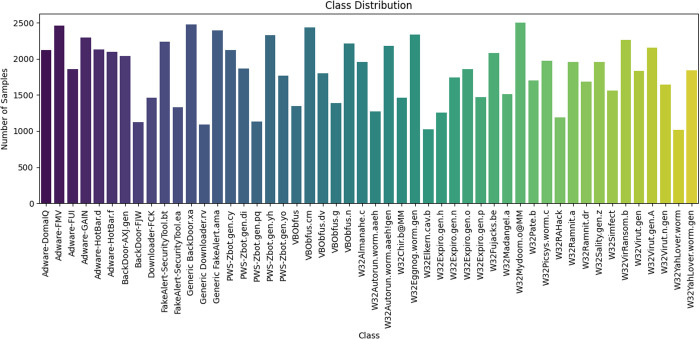
The number of samples for each malware class.

For data pre-preparation, VirusTotal is utilized to gather detailed behavioral data about Windows files. VirusTotal works by running the file in a secure, isolated environment, such as a virtual machine or sandbox, allowing it to execute as it would on a regular computer. This setup ensures that any potentially harmful actions performed by the file are contained and don’t affect real systems. Furthermore, for behavioral data, scraping of file contains multiple steps like, 1) Upload the File First; the file is uploaded to VirusTotal, where it is prepared for analysis. VirusTotal runs multiple checks to identify the file type and ensure it is ready for behavioral monitoring. In the second step, the file is executed in a controlled sandbox environment. This simulated environment mimics a real system, allowing the file to perform actions like modifying files, altering the registry, starting new processes, or making network connections. The third step is monitors of files tasks, where VirusTotal closely monitors every action the file takes. It logs changes to system resources, such as new files created, registry keys modified, or network activity detected. It also looks for suspicious actions like disabling security tools or communicating with unknown external servers. VirusTotal generates a comprehensive behavior report. This report outlines all the activities the file performed while running. It highlights any behavior that matches common malware patterns, such as unauthorized access to system settings or attempts to spread across a network. The file through Macafee antivirus engines gathers results from various security tools. This helps cross-check the file’s behavior with known malware signatures and techniques. Macafee antivirus engines generates 50 classes of different signatures. In addition, each class contains a varying number of data points, ranging from a minimum of 1,000 to a maximum of 2,500 it can be shown in Fig 3. The group of classes and subclass descriptions is presented in Table 2. The proposed work utilizes 82 features extracted from 50,000 McAfee antivirus behavioral logs. These features include registry files, various file system changes, network activities, and other relevant data. Seventy percent of the data is used for model training, while the remaining 30% is used for model validation.

**Table 2:** Grouping the malware classes and sub-classes.

Category	Classes
Adware	Adware-DomaIQ, Adware-FMV, Adware-FUI, Adware-GAIN, Adware-HotBar.d, Adware-HotBar.f
BackDoor	BackDoor-AXJ.gen, BackDoor-FJW, Generic BackDoor.xa
Downloader	Downloader-FCK, Generic Downloader.rv
FakeAlert	FakeAlert-SecurityTool.bt, FakeAlert-SecurityTool.ea, Generic FakeAlert.ama
PWS-Zbot (Password Stealer)	PWS-Zbot.gen.cy, PWS-Zbot.gen.di, PWS-Zbot.gen.pq, PWS-Zbot.gen.yh, PWS-Zbot.gen.yo
VBObfus (Obfuscation)	VBObfus, VBObfus.cm, VBObfus.dv, VBObfus.g, VBObfus.n
W32 Family	W32Almanahe.c, W32Autorun.worm.aaeh, W32Autorun.worm.aaeh!gen, W32Chir.b@MM, W32Eggnog.worm.gen, W32Elkern.cav.b, W32Expiro.gen.h, W32Expiro.gen.n, W32Expiro.gen.o, W32Expiro.gen.p, W32Fujacks.be, W32Madangel.a, W32Mydoom.o@MM, W32Pate.b, W32Pate.c, W32Picsys.worm.c, W32RAHack, W32Ramnit.a, W32Ramnit.dr, W32Sality.gen.z, W32Simfect, W32VirRansom.b, W32Virut.gen, W32Virut.gen.A, W32Virut.n.gen, W32YahLover.worm, W32YahLover.worm.gen

By extracting the behavioral data of Windows files through VirusTotal, it is easy to understand how potential malware operates and gain valuable insights into the threat level of suspicious files. Table 3 shows the information extraction from file for understanding the behavior information to classify the malware.

**Table 3:** Encoding API and behavior features types, and description.

Category	API #	Code	APIs
Hooking	0	HK	No APIs
Network	4	NT01-NT04	UDP: 23.99.222.162:123, HTTP: No APIs, DNS: No APIs, TCP: No APIs
Service	0	SR01-SR06	No services controlled, opened, created, started, opened-managers, or deleted
Process	6	PR01-PR06	Created: jqiwnhbgug.exe, gnfsmcvlptewkob.exe, bwxwpoig.exe, ptbsgebyuhnnj.exe, C:\Program Files\WORDPAD.EXE, C:\WINDOWS\system32\bwxwpoig.exe
Extra	2	EX01-EX02	IsDebuggerPresent, DeviceIoControl
Windows	1	WD01	Searched: Shell_TrayWnd
Runtime DLLs	22	DL01-DL22	kernel32.dll, advapi32.dll, comctl32.dll, comdlg32.dll, gdi32.dll, mpr.dll, ole32.dll, oleaut32.dll, shell32.dll, user32.dll, version.dll, winmm.dll, wsock32.dll, uxtheme.dll, setupapi.dll, rpcrt4.dll, netapi32, apphelp.dll, userenv.dll, clbcatq.dll, winspool.drv, c:\windows\system32\fontsub.dll
Mutex	7	MX01-MX07	ShimCacheMutex, CTF.LBES.MutexDefaultS, CTF.Compart.MutexDefaultS, CTF.Asm.MutexDefaultS, CTF.Layouts.MutexDefaultS, CTF.TMD.MutexDefaultS, CTF.TimListCache.MutexDefaultS
Filesystem	10	FS01-FS10	Opened: \PIPE\lsarpc, MountPointManager, C:\82eb46f80a5ffcc0e11e449909c23d86e6ea8d1d1bac3c2f9a391630475b6c49, WORDPAD.EXE, TASKMAN.EXE, etc.
Registry	0	RG01	No registry keys set or deleted

### 4.2. Feature extraction

BERT, or Bidirectional Encoder Representations from Transformers, is a transformer-based model designed to understand text by taking into account both the left and right context of every word in a sentence. This bidirectional nature allows BERT to understand relationships and dependencies between tokens (words) that other models might miss. In this work, malware behavior logs are treated as textual sequences and use BERT to model the dependencies between different actions. For example, if a malware sample accesses a specific file, creates a process, and makes a network request, BERT can learn the relationship between these actions to classify the behavior as malicious or benign.

The first step in using BERT is tokenizing the input. The *BertTokenizer* is used to convert the sequence of malware actions into tokens. Each sequence starts with a [*CLS*] token (used for classification tasks) and ends with a [*SEP*] token (which separates different parts of the sequence). It can be shown as:


[CLSHK: No APIs, NT01: UDP connection, PR01: Created process, FS01: Accessed file [SEP]

BERT uses this tokenized input to process the behavior log and generate contextualized embedding for each token.

The powerful model that can capture complex relationships in textual data is named as ‘BERT’ especially for malware classification. It can effectively learn patterns in API calls that represent the behavioral signatures of malware. Here, it shows a description of how feature extraction from different categories of malware behavior and their associated API calls can be easily extracted through BERT. In malware, for the interception of system, API calls hooking is referred, allowing malware to identify and modify legitimate system processes. The API calls associated with hooking, such as *SetWindowsHookEx*, are tokenized and processed as part of a sequence using BERT. The relationship between these hooking APIs and other system interactions can be captured by BERT. For example, if malware hooks into displaying system and later accesses confidential files, the attention mechanism of BERT will highlight these connections, allowing understanding the broader context in which hooking takes place for the model.


"[CLSHooking SetWindowsHookEx detected [SEP] Process CreateProcess executed [SEP]"

BERT can extract the semantic relationship between the hooking API and subsequent system actions, providing usable features for classification.

Malware immediately communicates with external servers, including *HttpSendRequest* or *DnsQuery*, which can be detected by API calls. These network-related API calls including capturing the malware’s communication patterns and sequential inputs can be processed by BERT. For detecting malware that extracts data or communicates with command-and-control (C2) servers, this category is difficult. Extracted features can recognize when malware initiates connections by encoding network-related actions into BERT, how frequently it communicates and what protocols it uses.


"[CLSNetwork HttpSendRequest issued [SEP] DNS DnsQuery executed [SEP]"

**Fig 4:**
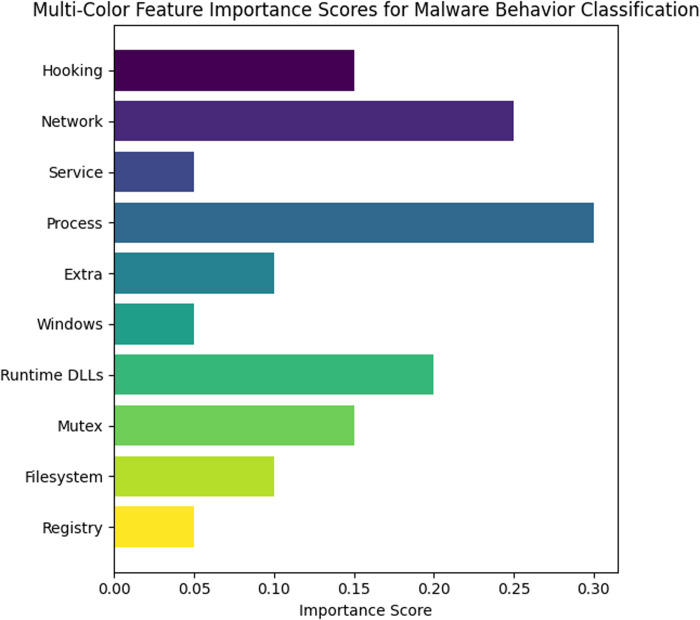
Feature Importance Scores for Malware Behavior Classification.

Process manipulation is a common tactic used by malware to execute its payload. By analyzing the API calls including “CreateProcess” and “TerminateProcess”, BERT can easily learn the sequence for creating and terminating a process. BERT can capture the dependencies between other system behaviors and recognize them by tokenizing these actions, providing insights into how malware manages processes. BERT’s contextual understanding enables it to extract suspicious patterns in process creation including malware terminating security-related processes or creating hidden processes. The interaction of malware is usually with reading, writing, or deleting in file system. BERT tokenizes the calls of file system API like DeleteFile and CreateFile, for extracting features related to file access patterns. For example, BERT can easily learn repetitive patterns and use them for differentiating between the features for detection of malware, if malware continuously reads or writes the system files. For establishing persistence, Registry modifications play vital role for malware behavior. BERT tokenizes API calls including ‘RegCreateKeyEx’ and ‘RegSetValueEx’ that are registry related to figure out and learn how malware changes the registry of system to handle settings or confirms it starts at boot. BERT extracts features that help in the classification of whether the malware is likely trying to change the setting of key; this can be done by encoding the API sequences. The ability of BERT is to understand the sequence and context of these actions to find out malware that handles the registry persistence on a system. Different malware behavior features importance score is shown in Fig 4.

After features extraction, the classification task is handled by adding a dense layer on top of the BERT model. The output corresponding to the [CLS] token is used as an aggregated representation of the entire sequence. This output is then passed through a fully connected layer with a *softmax* activation to produce the final classification. The classification layer is defined as:


$$\hat{\text{y}}=softmax(W_{h}h_{\left[CLS\right]}+b_{h})$$ (1)

where, $h_{\left[CLS\right]}$ is known as embedding for the [*CLS*], $W_{h}\;and\;b_{h}$ represent weights and biases, and $\hat{y}$ is the probability distribution of the predicted malware classes.

In this paper, the BERT model is fine-tuned using the labeled malware behavior dataset. The objective is to minimize the cross-entropy loss between the predicted class probabilities and the true labels. The loss function is defined as:


$$L=-\sum_{i=1}^{N}y_{i}\text{log}({\hat{y}}_{i})$$ (2)

where, $y_{i}$ are known as i^th^ sample of the label and ${\hat{y}}_{i},\;N$ are the predicted probability and the total number of samples of the dataset. During training, the weights of the BERT model are updated at the classification layer to optimize the classification performance.

## 5. Results and experiments

This research focuses on identifying malware behaviors by closely analyzing their memory-based features. The effectiveness of using these features for malware detection and classification was tested through two experiments. In the first experiment, six types of memory-based features are combined, while in the second, each type is evaluated individually. Using 10-fold cross-validation, the dataset was split into ten groups to ensure the classifier was trained on completely separate data from the testing phase.

To assess the effectiveness of the proposed method, a series of experiment is performed to find the robustness of the system. Furthermore, this article uses several class-specific metrics: accuracy, precision, recall, F1-measure, and overall accuracy, which reflects the model’s overall correctness. Recall (also referred to as positive predictive value) represents the likelihood that a sample belonging to class c is correctly classified. A recall of 1 indicates that the classifier consistently predicts whether an instance belongs to class c correctly. Precision measures how often the model’s prediction that a sample belongs to class c is correct. Low precision indicates that many samples are misclassified as being in class c. To get a more comprehensive understanding of the model’s performance, F-measure is used, a widely-used metric that calculates the harmonic mean of precision and recall. The F-measure ranges from 0 to 1, where a higher value indicates better performance, with 1 representing perfect classification.

Table 4 presents the final performance metrics after 100 epochs. It shows the training and validation losses, accuracy, precision, recall, and F1-score. At epoch 100, the training loss is 0.134 and the validation loss is 0.171. The model achieves an accuracy of 92.25%, a precision of 91.12%, a recall of 92.25%, and an F1-score of 91.22%. These metrics highlight the model’s effectiveness and overall performance after extensive training.

**Table 4:** Final performance metrics after 100 epochs.

Epoch	Training Loss	Validation Loss	Accuracy	Precision	Recall	F1-score
100	0.134	0.171	92.24%	91.11%	92.24%	91.22%

**Fig 5:**
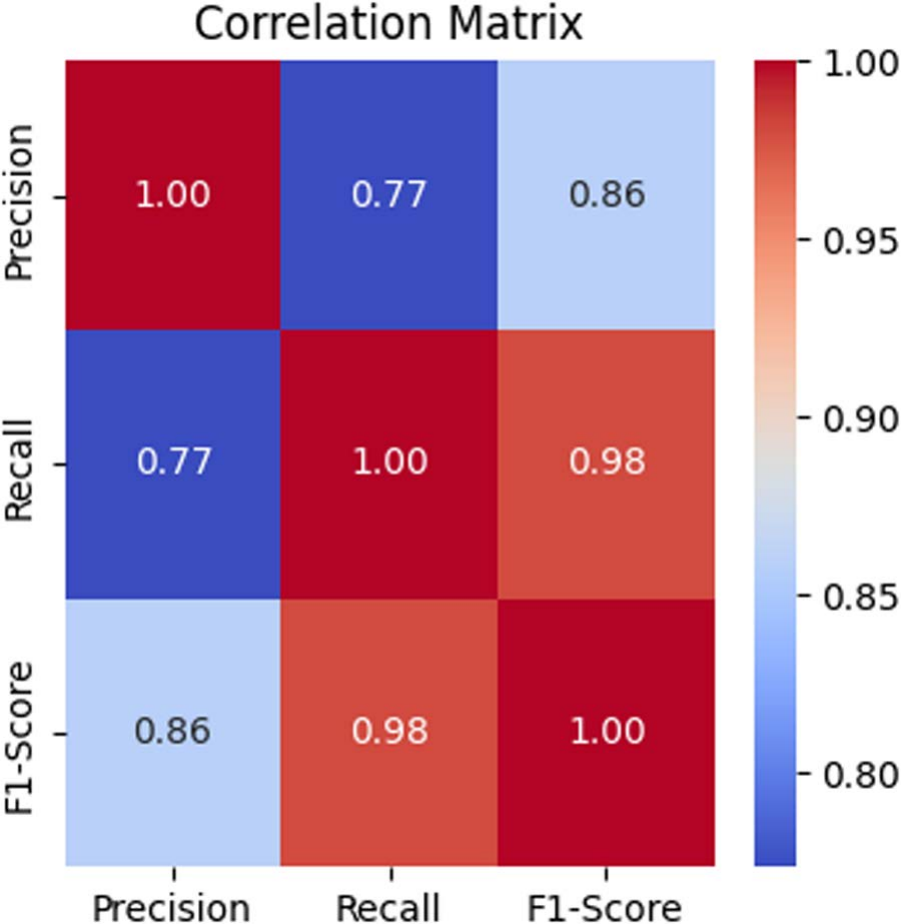
Correlation matrix of performance metrics calculated from class-specific.

In the upcoming experiment, each class is evaluated individually, without grouping similar classes as shown in Table 5. Different color representation is based on the precision 95%, 90%, 85%, and 80% and above 70%. The focus will be on malware families, where each family is treated as a distinct entity, allowing us to assess the performance of each class separately. By doing this, the aim is to measure the class-specific performance, providing a clearer understanding of how well each family is being identified. Additionally, a correlation matrix as shown in Fig 5 is drawn to analyze the relationships between the classes, offering insights into any potential overlaps or dependencies. This approach will give a more detailed and accurate picture of the classification performance across all malware families.

**Table 5:** Malware families and class-specific performance measure.

Class	Precision(%)	Recall(%)	F1-Score(%)
Adware-DomaIQ	1	97.14	98.55
Adware-FMV	96.77	96.77	96.77
Adware-FUI	73.53	98.04	84.03
Adware-GAIN	96.67	1	98.31
Adware-HotBar.d	1	98.61	99.3
Adware-HotBar.f	98.33	1	99.16
BackDoor-AXJ.gen	1	95.05	97.46
BackDoor-FJW	84.38	98.18	9076
Downloader-FCK	94.29	97.06	95.65
FakeAlert-SecurityTool.bt	86.96	62.5	72.73
FakeAlert-SecurityTool.ea	68.75	84.62	75.86
Generic BackDoor.xa	97.3	94.74	0.96.
Generic Downloader.rv	92.73	91.07	91.89
Generic FakeAlert.ama	64.52	57.14	60.61
PWS-Zbot.gen.cy	42.86	12.5	19.35
PWS-Zbot.gen.di	81.82	95.74	88.24
PWS-Zbot.gen.pq	73.33	81.48	77.19
PWS-Zbot.gen.yh	0.9	0.3	45.0.
PWS-Zbot.gen.yo	1	96.	97.96
VBObfus	88.89	94.12	91.43
VBObfus.cm	1	75.68	86.15
VBObfus.dv	67.65	92	77.97
VBObfus.g	68.49	89.29	77.52
VBObfus.n	1	06.25	11.76
W32Almanahe.c	1	95.52	97.71
W32Autorun.worm.aaeh	74.58	67.69	70.97
W32Autorun.worm.aaeh!gen	67.5	75	71.05
W32Chir.b@MM	94.23	96.08	95.15
W32Eggnog.worm.gen	1	98.18	99.08
W32Elkern.cav.b	97.78	1	98.88
W32Expiro.gen.h	53.77	68.67	60.32
W32Expiro.gen.n	0	0	0
W32Expiro.gen.o	27.78	5	35.71
W32Expiro.gen.p	0	0	0
W32Fujacks.be	91.57	87.36	89.41
W32Madangel.a	96.55	87.5	91.8
W32Mydoom.o@MM	98.67	1	99.33
W32Pate.b	86.81	99.03	92.52
W32Picsys.worm.c	1	99.06	99.53
W32RAHack	99.11	1	99.55
W32Ramnit.a	94.01	86.74	90.23
W32Ramnit.dr	75	88.24	81.08
W32Sality.gen.z	89.5	98.17	93.64
W32Simfect	89.53	93.9	91.67
W32VirRansom.b	97.27	99.53	98.39
W32Virut.gen	80.36	91.84	85.71
W32Virut.gen.A	1	37.5	54.55
W32Virut.n.gen	97.25	97.38	97.32
W32YahLover.worm	96.97	88.89	92.75
W32YahLover.worm.gen	76.67	92.0	83.64

In this experiment, the top five highest and lowest performing classes are examined based on their F1-Scores as shown in Table 6. The highest performing classes include W32RAHack with an F1-Score of 99.55%, W32Picsys.worm.c at 99.53%, W32Mydoom.o@MM scoring 99.33%, Adware-HotBar.d at 99.3%, and Adware-HotBar.f with 99.16%. These classes demonstrated strong detection accuracy and balanced precision and recall. Conversely, the lowest performing classes showed significant challenges. W32Expiro.gen.n, W32Expiro.gen.p, and W32Pate.c all had an F1-Score of 0, indicating no successful detections. PWS-Zbot.gen.cy had a modest score of 19.35% and W32Expiro.gen.o scored 35.71%, indicating poor overall performance in detection.

**Table 6:** Top five highest and lowest performing classes based on their F1-Scores.

Highest Classes by F1		Highest Classes by F1	
Class	F1-Score (%)	Class	F1-Score (%)
W32RAHack	99.55	**W32Expiro.gen.n**	0
W32Picsys.worm.c	99.53	**W32Expiro.gen.p**	0
W32Mydoom.o@MM	99.33	**W32Pate.c**	0
Adware-HotBar.d	99.3	**PWS-Zbot.gen.cy**	19.35
Adware-HotBar.f	99.16	**W32Expiro.gen.o**	35.71

In this experiment, the malware classification system’s performance is evaluated across various classes using precision, recall, and F1-Score as metrics as shown in Table 7. The results are presented in a class-wise manner, highlighting the detection accuracy for each malware category. For instance, Adware exhibited excellent performance with a precision of 93.7%, recall of 98.3%, and an F1-Score of 96.3%. Similarly, BackDoor and Downloader achieved high scores, reflecting strong detection capabilities. However, classes like FakeAlert (F1-Score: 70.7%) and PWS-Zbot (F1-Score: 70.1%) demonstrated weaker performance, revealing areas where the detection system struggles.

**Table 7:** Group-wise malware classification average results.

Class	Precision (%)	Recall (%)	F1-Score (%)
Adware	93.7	98.3	96.3
BackDoor	90.7	96.1	93.3
Downloader	93.5	94.1	93.8
FakeAlert	73.4	68.6	70.7
PWS-Zbot	72.3	69.7	70.1
VBObfus	82.5	75.5	76.8
W32	87.5	85.8	85.9

**Fig 6:**
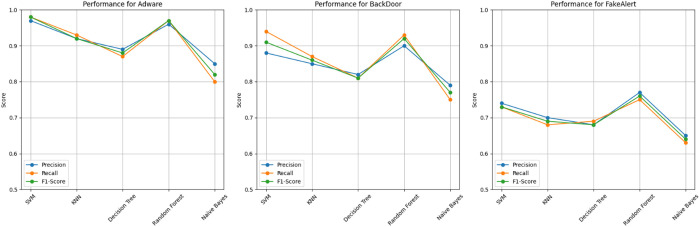
Graphical representation of per-classifier by class category.

Table 8 presents a comparative analysis of classifier performance across top 3 malware categories, utilizing precision, recall, and F1-Score metrics and a graphical representation in Fig 6. Classifiers tested include SVM, KNN, Decision Tree, Random Forest, and Naïve Bayes. For Adware, SVM achieves the highest F1-Score (0.98), while Random Forest also demonstrates robust performance (F1-Score: 0.97). Conversely, Naïve Bayes yields the lowest results for most categories, particularly in FakeAlert, with an F1-Score of 0.64. In the BackDoor category, Random Forest again outperforms other classifiers with an F1-Score of 0.92, indicating its consistency. This comparative table highlights classifier strengths and weaknesses across categories, guiding model selection and refinement.

**Table 8:** Per-Classifier Performance by Class Category.

Category	Metric	SVM	KNN	Decision Tree	Random Forest	Naïve Bayes
**Adware**	Precision	0.97	0.92	0.89	0.96	0.85
	Recall	0.98	0.93	0.87	0.97	0.8
	F1-Score	0.98	0.92	0.88	0.97	0.82
**BackDoor**	Precision	0.88	0.85	0.82	0.9	0.79
	Recall	0.94	0.87	0.81	0.93	0.75
	F1-Score	0.91	0.86	0.81	0.92	0.77
**FakeAlert**	Precision	0.74	0.7	0.68	0.77	0.65
	Recall	0.73	0.68	0.69	0.75	0.63
	F1-Score	0.73	0.69	0.68	0.76	0.64

Table 9 shows the results of using BERT feature vectors with different classifiers, comparing their performance based on precision, recall, and F1-Score. SVM performs the best overall, with a precision of 0.9112 and an F1-Score of 0.9122, followed closely by Random Forest (F1-Score: 0.90). KNN also performs fairly well with an F1-Score of 0.83. However, Decision Tree and Naïve Bayes show lower performance, with F1-Scores of 0.80 and 0.76, respectively. This comparison highlights SVM and Random Forest as the most effective classifiers when using BERT features for this task.

**Table 9:** BERT features vector classification results on different classifiers.

Classifier	Precision	Recall	F1-Score
SVM	0.9112	0.9225	0.9122
KNN	0.82	0.85	0.83
Decision Tree	0.79	0.81	0.80
Random Forest (RF)	0.90	0.91	0.90
Naïve Bayes	0.75	0.77	0.76

The above experiments show that SVM and Random Forest consistently deliver strong performance. For Adware, SVM achieved an F1-Score of 0.98, while Random Forest performed equally well. Naïve Bayes showed weaker results, particularly for FakeAlert, with an F1-Score of 0.64. These findings suggest that when paired with effective classifiers, behavior-based analysis significantly enhances malware detection accuracy.

### 5.1. Discussion

A comparative analysis of two different types of features is presented in Table 10. The classification performance based on manually extracted features from malware behavioral logs and BERT contextual features is compared across five classifiers: SVM, RM, KNN, DT, and Naive Bayes. Performance is evaluated using F1-score and precision. The results show that, overall, BERT features outperform manual features. SVM achieves the highest performance, with a precision of 0.9122 using BERT features and 0.83 with manual features, as well as an F1-score of 0.9122 for BERT and 0.83 for manual features.

**Table 10:** Comparative Analysis of BERT features with Manual features on different classifiers.

Classifier	Precision (Manual)	Precision (BERT)	F1-Score (Manual)	F1-Score (BERT)
SVM	0.83	0.91	0.83	0.91
KNN	0.80	0.90	0.81	0.90
Decision Tree	0.75	0.82	0.76	0.83
Random Forest (RF)	0.72	0.79	0.73	0.80
Naïve Bayes	0.68	0.75	0.69	0.76

To evaluate the performance of the proposed work, a detailed comparison with other widely used techniques is presented in Table 11. This comparison highlights the techniques used, types of features, datasets, and overall results. In [ref], a publicly available dataset was used on MalBERTv2, a specialized BERT model designed for malware detection and analysis, which achieved 99% accuracy. However, this technique focuses solely on static analysis and is limited to source code and top-ranked files as input. In [ref], the authors employed a BERT model on an Android app manifest dataset and achieved promising accuracies around 97% for malware/Goodware classification and 93% for malware family classification. However, this approach focuses primarily on mobile malware detection. In another study [ref], the authors applied FastText and BERT to an API call sequence dataset, achieving 95.20% accuracy with FastText and 88.06% with BERT. However, this method primarily relies on the sequence of API calls, which can be affected by sequence length, ordering issues, and evasion techniques.

**Table 11:** Comparison of the proposed model with other state-of-the-art Techniques.

Ref	Technique/ Model	Features Used	Dataset	Results
[24]	MalBERTv2 (BERT)	Source Code, Top-ranked files	Public Malware/Goodware Datasets	82% − 99% (F1 score)
[25]	BERT	Android App Manifests	AndroZoo (265k Apps)	97% accuracy on Malware/Goodware, 93% on Family Classification
[26]	fastText, BERT	API Call Sequence, Behavioral Information	Benign Software, 2500 Malware Samples, CSDMC, APIMDS	Classification accuracy for fast text is 95.40% and 88.06% for BERT. Similarly, Detection accuracy for fast text is 99.86% and 97.11% for BERT.
Proposed Study	BERT, SVM, RF, Naive Bayes	Behavioral logs (File mods, registry changes, network connections)	Self-created dataset VirusTotal (Various Malware)	SVM achieve F1-Score 0.98 on Adware, 0.91 on BackDoor and 0.64 on FakeAlert

In comparison, this proposed work uses behavioral log files from VirusTotal, specifically targeting several malware families, which highlights the model’s practical effectiveness. It includes dynamic aspects of malware behavior such as registry modifications, file system changes, and network events, making it a more comprehensive and robust solution. The work introduces a novel malware detection technique that uses BERT to extract meaningful features from behavior logs features often overlooked by traditional statistical methods. By integrating BERT with behavioral patterns, this approach effectively overcomes the limitations of signature-based detection, significantly improving the identification of new and evolving threats.

## 6. Conclusion

In conclusion, malware classification remains a significant challenge due to the ever-evolving nature of malicious software. Traditional methods, such as signature-based detection, are often inadequate for identifying new and sophisticated threats, while static analysis can miss subtle malware characteristics. This underscores the importance of behavior-based analysis, which examines how files perform in a real-world environment to uncover hidden dangers. To improve detection accuracy, the BERT model is leveraged, originally designed for text processing, to extract significant features from the behavior logs. These features are then evaluated using several classifiers, including SVM, KNN, Decision Tree, Random Forest, and Naïve Bayes, to determine if a file is malicious. A behavior-based approach, which combines BERT for feature extraction with robust classifiers, offers a more accurate and reliable method for malware detection. The effectiveness of SVM and Random Forest highlights their suitability for this task, demonstrating significant improvements in identifying and classifying malicious software.

## Supporting information

S1 File Supporting_Information_Files. (RAR)
